# Large-scale dose evaluation of deep learning organ contours in head-and-neck radiotherapy by leveraging existing plans

**DOI:** 10.1016/j.phro.2024.100572

**Published:** 2024-03-28

**Authors:** Prerak Mody, Merle Huiskes, Nicolas F. Chaves-de-Plaza, Alice Onderwater, Rense Lamsma, Klaus Hildebrandt, Nienke Hoekstra, Eleftheria Astreinidou, Marius Staring, Frank Dankers

**Affiliations:** a Division of Image Processing (LKEB), Department of Radiology, Leiden University Medical Center, Leiden 2333 ZA, The Netherlands; b HollandPTC consortium – Erasmus Medical Center, Rotterdam, Holland Proton Therapy Centre, Delft, Leiden University Medical Center (LUMC), Leiden and Delft University of Technology, Delft, The Netherlands; c Department of Radiation Oncology, Leiden University Medical Center, Leiden 2333 ZA, The Netherlands; d Computer Graphics and Visualization Group, EEMCS, TU Delft, Delft 2628 CD, The Netherlands

**Keywords:** Automated plan optimization, Auto contouring, Dose impact, Robot process automation, Automated plans

## Abstract

**Background and purpose:**

Retrospective dose evaluation for organ-at-risk auto-contours has previously used small cohorts due to additional manual effort required for treatment planning on auto-contours. We aimed to do this at large scale, by a) proposing and assessing an automated plan optimization workflow that used existing clinical plan parameters and b) using it for head-and-neck auto-contour dose evaluation.

**Materials and methods:**

Our automated workflow emulated our clinic’s treatment planning protocol and reused existing clinical plan optimization parameters. This workflow recreated the original clinical plan ($P_{\mathrm{OG}}$) with manual contours ($P_{\mathrm{MC}}$) and evaluated the dose effect ($P_{\mathrm{OG}}-P_{\mathrm{MC}}$) on 70 photon and 30 proton plans of head-and-neck patients. As a use-case, the same workflow (and parameters) created a plan using auto-contours ($P_{\mathrm{AC}}$) of eight head-and-neck organs-at-risk from a commercial tool and evaluated their dose effect ($P_{\mathrm{MC}}-P_{\mathrm{AC}}$).

**Results:**

For plan recreation ($P_{\mathrm{OG}}-P_{\mathrm{MC}}$), our workflow had a median impact of 1.0% and 1.5% across dose metrics of auto-contours, for photon and proton respectively. Computer time of automated planning was 25% (photon) and 42% (proton) of manual planning time. For auto-contour evaluation ($P_{\mathrm{MC}}-P_{\mathrm{AC}}$), we noticed an impact of 2.0% and 2.6% for photon and proton radiotherapy. All evaluations had a median ΔNTCP (Normal Tissue Complication Probability) less than 0.3%.

**Conclusions:**

The plan replication capability of our automated program provides a blueprint for other clinics to perform auto-contour dose evaluation with large patient cohorts. Finally, despite geometric differences, auto-contours had a minimal median dose impact, hence inspiring confidence in their utility and facilitating their clinical adoption.

## Graphical abstract

**Figure:**
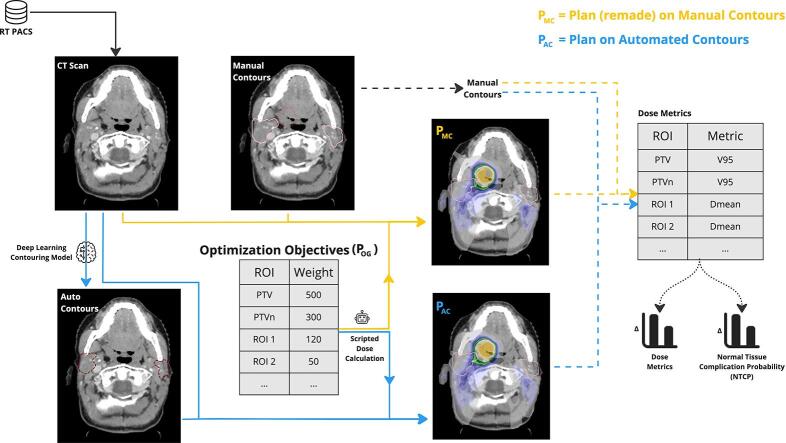
Workflow for automated plan optimization and use-case of evaluating the effect of automated contours on dose. By reusing original plan (P_OG_) parameters, we made a plan for both the manual contours (P_MC_) and automated contours(P_AC_), shown with yellow and blue colors respectively. Dashed lines indicate the evaluation workflow where both doses were evaluated on the manual contours. Pink, maroon and orange contours are used to represent the manual, automated and PTV (DL1) contours respectively. Finally, we used manual contours to compute dose metrics and normal tissue complication probability (NTCP) models and compare all plans.

## 1 Introduction

Manual contouring of organs-at-risk (OAR) in radiotherapy is a time and resource-demanding task [1], [2], [3], especially in head-and-neck cancer due to a large OAR count [4]. Moreover, it is plagued by inter- and intra-annotator variability [5], [6], [7], [8] and hence there is a need for automation. In the last few years, availability of deep learning-based commercial tools have reduced the barriers for clinics to implement auto-contouring technology in daily practice. However, these tools may produce erroneous contours due to poor contrast, organ deformations, surgical removal of an organ or when tested on different patient cohorts [9]. Such cases may potentially lead to commercial providers providing updates to the underlying deep learning models. Thus, as deep learning auto-contouring tools are increasingly adopted in clinics, with the potential for future updates to models, there is a growing need to benchmark them, preferably at large-scale and in an automated manner.

As deep learning-based auto-contouring methods for head-and-neck OARs have been shown to offer satisfactory geometric performance [10], [6], the next step is to evaluate their dose impact [11]. However, we observed that dose-based studies on auto-contours tend to use either smaller (≤20) [12], [13], [14], [15], [16], [17], [18] or medium-sized (⩽40) [19], rather than larger [20] datasets. Studies using larger datasets simply superimpose the automated contours on the clinical dose [20] which does not fully replicate the treatment planning process. Conversely, studies using smaller or medium-sized test datasets either made manual plans [14], [17], [18], [19], used knowledge-based planning [13], a template approach [12] or a priori multi-criteria optimization (MCO) [15], [16]. Since smaller datasets may be affected by sampling bias, there is a need to perform dose analysis with a larger patient cohort. However, a manual approach to plan optimization is simply not scalable. Moreover, existing automated approaches [13], [12], [15], if not already clinically implemented, require additional skills and resources. Therefore, there is a need for an automated approach to treatment planning that can be done at a large scale and also leverages existing clinical knowledge and work.

Thus, our contribution was to propose and assess a plan optimization method for retrospective studies that is scalable due to its automated nature and easily implementable due to the use of existing clinical resources (i.e., knowledge, tools and optimization parameters). We then used this approach in a use case to quantify auto-contour-induced dose effects for head-and-neck photon and proton radiotherapy.

## 2 Materials and methods

### 2.1 Data acquisition

Our dataset consists of 100 head-and-neck cancer patients, of which 70 had clinical plans made for photon therapy, while 30 had proton plans, at Leiden University Medical Center (Leiden, The Netherlands) from 2021 to 2023. Patients were treated for either oropharyngeal (71) or hypopharyngeal (29) cancers with cancer stages T1-4, N0-3 and M0. 92 patients were treated with curative intent, i.e., 7000 cGy to the primary tumor, while others were prescribed 6600 cGy due to their post-operative nature. Details about CT scans used in planning are written in Supplementary Material A. The study was approved by the Medical Ethics Committee of Leiden, The Hague, Delft (G21.142, October 15, 2021). Patient consent was waived due to the retrospective nature of the study.

### 2.2 Automated contours

For automated contouring, a commercial deep learning model from RayStation-10B (RaySearch Labs, Sweden) – “RSL Head and Neck CT” (v1.1.3) was used. A subset of the OARs which were used clinically for treatment planning were auto-contoured – Spinal Cord, Brainstem, Parotid (L/R), Submandibular (L/R), Oral Cavity, Esophagus, Mandible and Larynx (Supraglottic). See Supplementary Material B for additional details.

### 2.3 Treatment planning protocol

We used volumetric modulated arc therapy (VMAT) to generate a photon plan using a 6MV dual arc beam. The elective and boost Planning Target Volumes (PTV), henceforth referred as DL1/DL2 (dose level 1/2) were prescribed 5425 cGy/7000 cGy in 35 fractions. For post-operative patients, our clinic prescribed 5280 cGy/6600 cGy in 33 fractions instead. Planning was done such that at least 98% of DL1 and DL2 volumes received 95% of the prescribed dose (V_95%_) and also by keeping D_0.03*cc*_ for DL2 below 107% of the prescribed dose.

Proton plans consisted of six beam intensity modulated proton therapy (IMPT). Planning was done such that V_95%_ ⩾98% for DL1/DL2 and D_2%_ ⩽107% for DL2 of the Clinical Target Volume (CTV) in a 21-scenario robust optimization with 3 mm setup and 3% proton range uncertainty. For robust evaluation of CTV DL1/DL2 we instead use 28-scenarios and test the voxel-wise minimum (vw-min) plan such that its V_94%_ ⩾98% [22] and voxel-wise maximum (vw-max) of $D_{2\%}⩽107\%$.

### 2.4 Automated treatment planning

To make our automated program, a four-step script [23], [24], [25] was created which uses manually defined beam settings and objective weights from the clinical plan (more details in Supplementary Material C). This approach is also referred as robot process automation (RPA) [26], a process wherein a program emulates a human.

In summary, for step 1, we began with an objective template i.e., a class solution with a standard set of weights that focuses on targets and the body contour. Step 2 then added dose-fall-off (DFO) objectives for organs which is the distance over which a specified high dose falls to a specified low dose. In step 3, we introduced equivalent uniform dose (EUD) objectives [27] on the OARs. Manual planning for the EUD objective involves iteratively fine-tuning its parameters. Since only the parameters of the last iteration were available to us, we instead followed a single-step optimization for this objective. Finally, in step 4, we used patient-specific control structure contours to reduce OAR dose or sculpt the dose to the targets. In the last step, we also updated any other weights the treatment planner might have changed compared to the objective template. Note, these final weight updates were asynchronous to manual planning, since we did not know when these weights were updated in the aforementioned process. Note that each of the above steps underwent four optimization cycles.

**Fig. 1:**
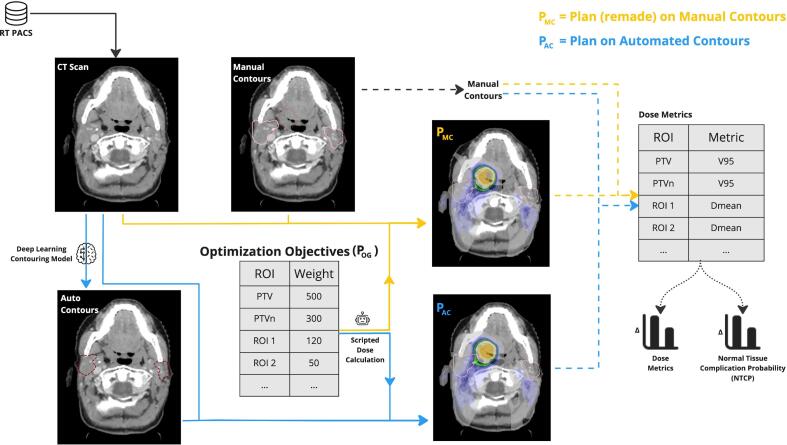
Workflow for automated plan optimization and use-case of evaluating the effect of automated contours on dose. By reusing original plan ($P_{\mathrm{OG}}$) parameters, we made a plan for both the manual contours ($P_{\mathrm{MC}}$) and automated contours($P_{\mathrm{AC}}$), shown with yellow and blue colors respectively. Dashed lines indicate the evaluation workflow where both doses were evaluated on the manual contours. Pink, maroon and orange contours are used to represent the manual, automated and PTV (DL1) contours respectively. Finally, we used manual contours to compute dose metrics and normal tissue complication probability (NTCP) [21] models and compare all plans.

Using our automated program, we made two plans – 1) a plan optimized on manual contours (P_*MC*_) and 2) a plan optimized on automated contours (P_*AC*_) as shown in Fig. 1. For the targets, elective lymph nodes, and OARs not available in the auto-contouring model we used manual contours which were used clinically for the original plan (P_*OG*_). The plans were made using the Python 3.6 scripting interface of the Treatment Planning System (TPS) of RayStation. The scripts for this work are available at  https://github.com/prerakmody/dose-eval-via-existing-plan-parameters.

### 2.5 Geometric evaluation

We used volumetric and surface distance metrics like Dice Coefficient, Hausdorff Distance 95% (HD95) and Mean Surface Distance (MSD) to evaluate our contours. Moreover, we also evaluated Surface DICE (SDC) with a margin of 3 mm to gain insight into contour editing time requirements [28].

### 2.6 Dose and NTCP evaluation

Given that our plans – $P_{\mathrm{OG}},P_{\mathrm{MC}}$ and $P_{\mathrm{AC}}$ have differences in the way they were created, we need to compare them. Metrics relevant to OARs were calculated and plans were compared in the following manner:

(1) $$\Delta D_{x}=D_{x,p1}-D_{x,p2}\text{.}$$

Here, x refers to the OAR for which we calculated a dose metric D and then compared it between any pair of plans p1 and p2. Here, D can refer to $D_{0.03\mathrm{cc}}$ (Spinal Cord, Brainstem), $D_{\mathrm{mean}}$ (Parotid, Submandibular, Oral Cavity, Larynx (Supraglottic), Esophagus) or $D_{2\%}$ (Mandible).

For normal tissue complication (NTCP) probability [21] evaluation, we used a similar approach:

(2) $$\Delta {\mathrm{NTCP}}_{d}={\mathrm{NTCP}}_{d,p1}-{\mathrm{NTCP}}_{d,p2},$$

where d refers to either Xerostomia or Dysphagia with a grade ⩾2 or ⩾3.

For the above $\Delta D_{x}$ (dose) and $\Delta {\mathrm{NTCP}}_{d}$ values, we performed a Wilcoxon signed-rank test (p ⩽0.05 is considered a significant difference) to evaluate if the differences between plans are significant.

## 3 Results

### 3.1 Geometric evaluation

**Fig. 2:**
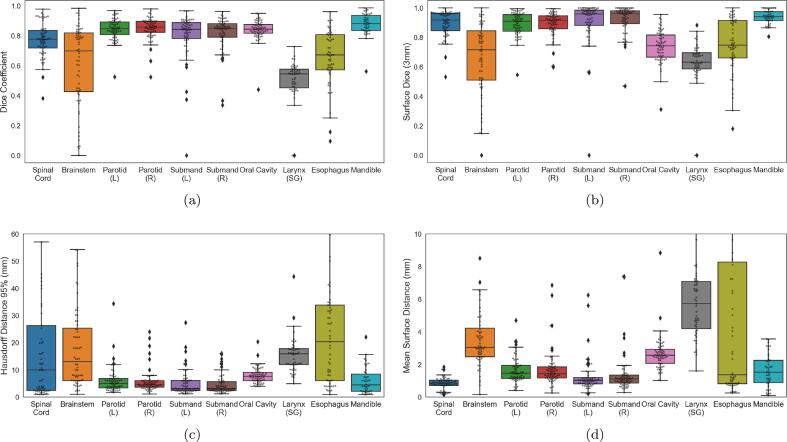
Box plots showing geometric (a) and surface metrics (b–d) for all our patients. The scatter points indicate the metric values for each patient.

Fig. 2 shows five organs (Spinal Cord, Parotids, Submandibulars, Oral Cavity, Mandible) had a median DICE ⩾0.78 (with additional summary measures tabulated in Supplementary Material B). In Fig. 2b we observed that in general the surface DICE values for the OARs are higher than their DICE values, except for the oral cavity. Fig. 2c and Fig. 2d shows that HD95 and MSD had trends similar to DICE in Fig. 2a. OARs with a median DICE ⩾0.8 had their median HD95 less than 7.7 mm and their median MSD less than 2.6 mm. The spinal cord had DICE values that are better than brainstem, but its HD95 range was as long as brainstem.

### 3.2 Dose evaluation

**Fig. 3:**
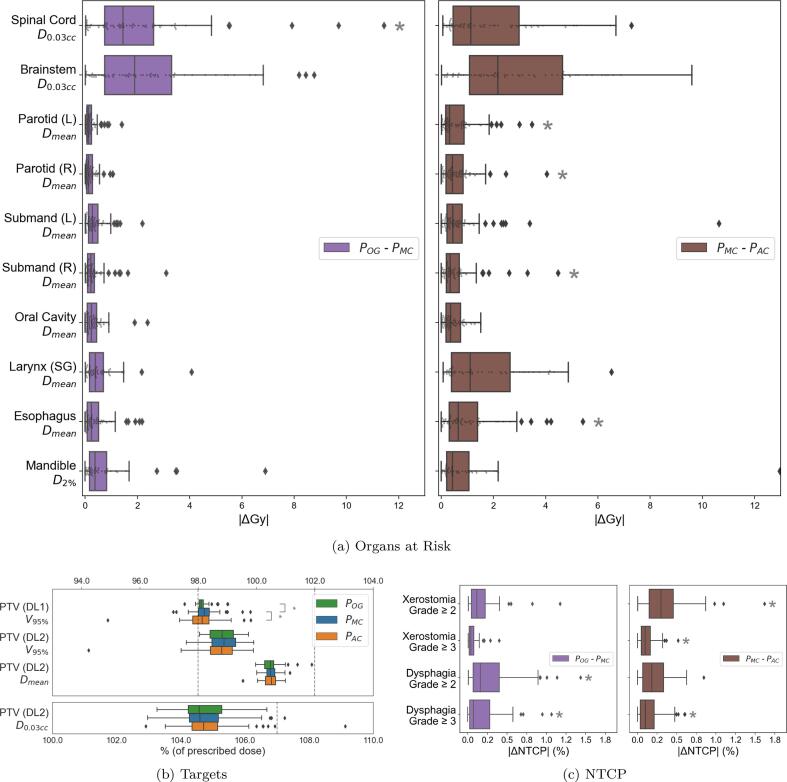
Dose metrics for the original (i.e., clinical) photon plans ($P_{\mathrm{OG}}$) as well as plans (re) made on manual ($P_{\mathrm{MC}}$) and automated ($P_{\mathrm{AC}}$) contours using an automated program. $P_{\mathrm{OG}}-P_{\mathrm{MC}}$ shows the dose effect of the proposed planning process, while $P_{\mathrm{MC}}-P_{\mathrm{AC}}$ shows the effect of using auto-contours. Here  represents a p-value ⩽0.05. In a) we see the difference in the dose metric of each OAR when comparing across plans. The plots in b) show us the metrics for the targets, while c) shows us the difference in NTCP values.

The median absolute value of $P_{\mathrm{OG}}$ (original plan) - $P_{\mathrm{MC}}$ (automated plan using manual contours) was 0.27 Gy (1.0%), 1.66 Gy (4.6%) and 0.21 Gy (0.7%) for all, central nervous system (CNS), i.e., Brainstem and Spinal Cord and non-CNS organs, respectively. The same for $P_{\mathrm{MC}}$ - $P_{\mathrm{AC}}$ (automated plan using auto-contours) was 0.58 Gy (2.0%), 1.86 Gy (5.4%) and 0.46 Gy (1.6%), with metrics of individual organs in Fig. 3a listed in Supplementary Material D. Fig. 3b shows dose metrics for targets where, for $P_{\mathrm{MC}}$ and $P_{\mathrm{AC}}$, we achieved PTV (DL1) ($V_{95}$) ⩾98.0% for 76% and 60% of plans. However, 96% and 93% of $P_{\mathrm{MC}}$ and $P_{\mathrm{AC}}$ plans achieved PTV (DL1) ($V_{95}$) ⩾97.5%. For this metric, a statistically significant difference was observed between $P_{\mathrm{OG}}$ and $P_{\mathrm{MC}}$ as well as $P_{\mathrm{MC}}$ and $P_{\mathrm{AC}}$. Finally, Fig. 3c shows |ΔNTCP| results, where the maximum median across all toxicities was 0.3% (individual toxicity metrics in Supplementary Material E).

**Fig. 4:**
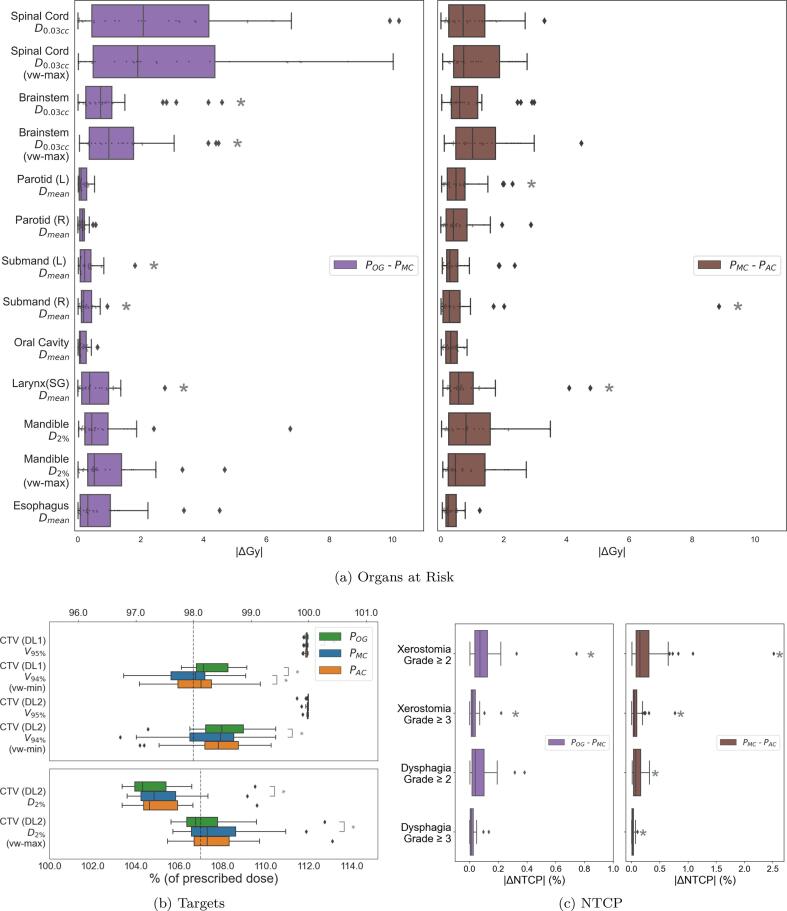
Dose metrics for the original proton plans ($P_{\mathrm{OG}}$) as well as plans (re) made on manual ($P_{\mathrm{MC}}$) and automated ($P_{\mathrm{AC}}$) contours using an automated program. $P_{\mathrm{OG}}-P_{\mathrm{MC}}$ shows the dose effect of the proposed planning process, while $P_{\mathrm{MC}}-P_{\mathrm{AC}}$ shows the effect of using auto-contours. Here  represents a p-value ⩽0.05. In a) we see the difference in the dose metric of each OAR when comparing across plans. The plots in b) show us the metrics for the targets, while c) shows us the difference in NTCP values.

For proton, $\left|P_{\mathrm{OG}}-P_{\mathrm{MC}}\right|$ had a median value of 0.33 Gy (1.5%), 1.13 Gy (11.5%) and 0.22 Gy (0.8%) for all, CNS and non-CNS organs, respectively. The same for $P_{\mathrm{MC}}-P_{\mathrm{AC}}$ was 0.48 Gy (2.6%), 0.75 Gy (6.9%) and 0.38 Gy (1.8%). Fig. 4b shows proton targets wherein 58% and 62% of $P_{\mathrm{MC}}$ and $P_{\mathrm{AC}}$ plans achieved PTV (DL1) (vw-min) ($V_{94}$) ⩾98.0%, while 82% and 80% achieved PTV (DL1) (vw-min) ($V_{94}$) ⩾97.5%. Similar to photon, a statistically significant difference was observed between $P_{\mathrm{OG}}$ and $P_{\mathrm{MC}}$ as well as $P_{\mathrm{MC}}$ and $P_{\mathrm{AC}}$. For |ΔNTCP| (Fig. 4c), the maximum median across all toxicities was 0.2%.

**Fig. 5:**
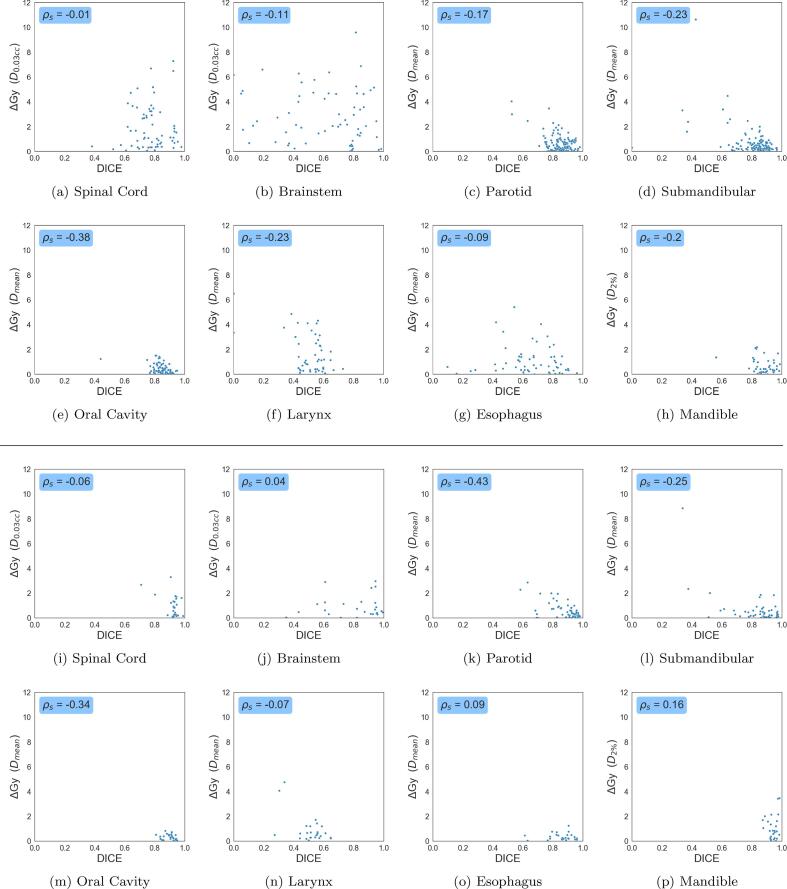
Scatter plots for eight organs-at-risk from the auto-contouring module. Here we plot the DICE (x-axis) against each organs absolute dose metric differences, i.e., $\left|P_{\mathrm{MC}}-P_{\mathrm{AC}}\right|$ (y-axis) for photon (a–h) and proton (i–p) radiotherapy.

A weak Spearman correlation coefficient between DICE and dose differences ($\left|P_{\mathrm{MC}}-P_{\mathrm{AC}}\right|$) was observed for CNS organs ($\left|\rho_{s}|⩽0.11\right)$, across both photon and proton (Fig. 5). Conversely, the Parotids, Submandibulars and Oral Cavity had relatively higher values ($-0.43⩽\rho_{s}⩽-0.17$). The remaining organs did not have similar correlations across both radiotherapy treatments.

Finally, our automated plan optimization took 45 min and 2.5 h of computer time, compared to 3 and 6 h of manual time (on average, as estimated by our clinic’s planners), for photon and proton, respectively.

## 4 Discussion

This work aimed at proposing and assessing an automated plan optimization workflow for retrospective studies that can be easily implemented by clinics due to its use of existing clinical resources. Unlike previous works [12], [13], [14], [15], [16], [17], [18], we performed this at large-scale and for both photon and proton radiotherapy. To replicate our approach, a clinic can simply use the scripting interface of their treatment planning system (TPS) and convert their planning process into a step-by-step approach. This requires minimal additional expertise (i.e., Python coding), for which many TPS solutions provide documentation. For head-and-neck radiotherapy, automated plans on manual contours ($P_{\mathrm{MC}}$) showed a negligible difference (i.e., median impact of 1.0% and 1.5% across organs), when compared to the original clinical plan ($P_{\mathrm{OG}}$) [29], [30]. Thus, the proposed evaluation process could serve as a springboard for clinics to validate an auto-contouring model, at large-scale, by simply reusing their existing plans. When using this program for the use case of head-and-neck auto-contour evaluation, the plan using auto-contours ($P_{\mathrm{AC}}$) had a low dose impact when compared to the plan using manual organ contours, for both photon (2.0%) and proton (2.6%) planning. Additionally, minuscule differences in NTCP values indicated that minor plan differences did not lead to large differences in long-term radiation-induced toxicity. This could potentially promote confidence in the community [31] to adopt auto-contouring to speed up clinical workflows.

**Fig. 6:**
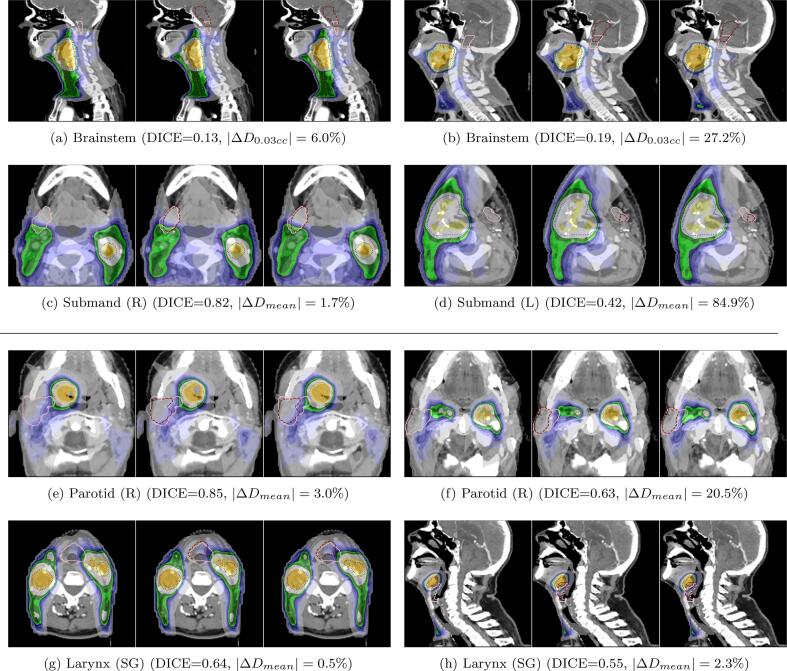
CT scans of photon (a–d) and proton (e–h) patients overlayed with a dose distribution as well as PTV (DL1) (orange), PTV (DL2) (blue), manual (pink) and automated (maroon) contours. Each example shows the $P_{\mathrm{OG}},P_{\mathrm{MC}}$ and $P_{\mathrm{AC}}$ plans from left to right. The dose metric in the sub-captions compares the absolute percentage difference of $P_{\mathrm{MC}}-P_{\mathrm{AC}}$. (For interpretation of the references to colour in this figure legend, the reader is referred to the web version of this article.)

For five out of eight OARs (i.e., Spinal Cord, Parotid, Submandibular, Oral Cavity and Mandible), the average DICE scores may be considered on par with previous work (≈0.8) [6], [10], [12] (see Supplementary Material B). A visual inspection of the remaining auto-contours, i.e., Larynx (SG), Brainstem (and by extension the Spinal Cord) (Fig. 6, Supplementary Material F) indicated that they had contouring protocols that differed from our clinic. Moreover, the auto-contouring model was trained on a different patient cohort, leading to additional contour differences with our clinical dataset. Finally, we chose to not perform any additional refinement on manual contours, since they were also used for making clinical plans ($P_{\mathrm{OG}}$) delivered to patients. For e.g. in the first row of Fig. 6, we see that only the caudal section of the Brainstem was annotated. Treatment planners find optimizing this section sufficient due to its potential for high dose from tumor proximity. The aforementioned reasons are why we noticed reduced measures for Larynx (SG), Brainstem and Spinal Cord in Fig. 2.

A critique of using unmodified manual contours may be that a lack of “gold-standard” contours will not give accurate geometric measures. Since our primary goal however was dose evaluation using existing clinical resources (i.e., unmodified manual contours), we proceed without any refinement. Also, in an auto-contouring dose evaluation scenario, it is already sufficient to know that plans made on auto-contours are equivalent to plans made on manual contours as seen in Fig. 3b (photon) and Fig. 4b (proton). Thus, our approach of using existing manual contours improves the ease-of-implementation of auto-contour dose evaluation studies and enables evaluation at large-scale.

To evaluate the quality of our automated plans, we first assessed target dose metrics. We use PTV (DL1) ($V_{95\%}$) for photon and CTV (DL1) ($V_{94\%}$) (vw-min) for proton, since planners prioritize them due to their difficulty. Hence it serves as a good benchmark for our automated plans. Results indicated that most of our plans (⩾93% for photon and ⩾80% for proton) were of near-clinical quality (i.e., ⩾97.5%). Those plans that did not strictly achieve clinical quality (i.e., ⩾98%) on the aforementioned metrics, had reduced dose coverage in either the most cranial or caudal slices. In a retrospective study for dose-evaluation of auto-contours, such a minor error will have a minimal effect on the dose metrics of organs we are interested in.

Fig. 4b shows that most proton plans, including $P_{\mathrm{OG}}$, tended to have hotspots, i.e., $D_{2\%}(\mathrm{vw}-\max)⩾107\%$, unlike most photon plans which did not, i.e., $D_{0.03\mathrm{cc}}⩽107\%$ (Fig. 3b). In our dataset, these proton plans were made for performing a plan comparison between photon and proton (via NTCP), according to the model-based selection [32]. If during proton treatment planning, the NTCP differences already indicated either a) high organ sparing or b) not sufficiently better organ sparing than photons, planners did not further optimize this plan. However, given that dose hotspots are quite small, they did not affect dose metrics for the auto-contoured organs in our study. Finally, differences in plans were also caused because the same plan optimization process when run twice, may lead to similar, but not exactly the same solution due to randomness in initialization.

Fig. 3 shows that of all the organs the Spinal Cord and Brainstem had wider boxplots for both $P_{\mathrm{OG}}-P_{\mathrm{MC}}$ and $P_{\mathrm{MC}}-P_{\mathrm{AC}}$. This is because the $\Delta D_{0.03\mathrm{cc}}$ metric is inherently more sensitive to dose changes than $\Delta D_{\mathrm{mean}}$. This is seen in the first row of Fig. 6 where similar DICE values for the Brainstem output vastly different dose differences. For proton (Fig. 4), we saw a similar trend for $P_{\mathrm{OG}}-P_{\mathrm{MC}}$, but not for $P_{\mathrm{MC}}-P_{\mathrm{AC}}$. This indicated that proton planning is more susceptible to workflow differences than contour differences of Brainstem and Spinal Cord, for our cohort of oro- and hypopharyngeal cancers, which are at a distance from these organs.

Fig. 3a, 3c (photon) and Fig. 4a, 4c (proton) show statistically significant differences, but from a clinical standpoint, the minor differences in organ dose metrics and ΔNTCP values may be clinically irrelevant.

Moving on to the effect of DICE on dose metric of organs (Fig. 5), one would expect that a decrease in DICE would lead to higher ΔcGy values for organs. This was true for the Parotids, Submandibulars (Fig. 6) and Oral Cavity across both photons and protons ($-0.43⩽\rho_{s}⩽-0.17$). The Brainstem and Spinal Cord showed poor correlation scores for both forms of radiotherapy, primarily due to the sensitive nature of the $D_{0.03\mathrm{cc}}$ metric. The Esophagus also showed low correlation, since, in many cases, it is caudally far away from the tumor regions for the patients in our cohort. The Larynx showed a high correlation for photon, but not for proton, which could be an effect of sample size. Finally, the Mandible, an organ with high DICE, showed opposite trends in photon and proton. Overall, we noticed that there was a low correlation between DICE and dose metrics.

This work was inspired by prior research on treatment plan scripting [24], [23] to scale-up dose evaluation for auto-contours. However, some plans were still not of the highest possible quality since our four-step replication of the clinical process is a close, but imperfect emulation of a treatment planners approach. Non-iterative EUD optimization (step 3), lack of synchrony in weight updates between the manual and automated approach (step 4), and re-use of control structures from $P_{\mathrm{OG}}$ to $P_{\mathrm{MC}}$ and $P_{\mathrm{AC}}$ (step 4), led to small deviations from the original planning process. These limitations cause $P_{\mathrm{MC}}$ and $P_{\mathrm{AC}}$ dose metrics to be imprecise which could potentially impact our results. For future work we would like to more closely mimic the optimization steps as well as consider control structures specific to each plan, rather than simply copying them.

To conclude, we showed an automated approach to plan creation for retrospective studies that was employed for the use-case of evaluating the dose impact of auto-contouring software, at scale. We hope our results showcasing low dose impact of auto-contours will inspire others to investigate and eventually use them in clinical settings.

## Funding

The research for this work was funded by Varian, a Siemens Healthineers Company, through the HollandPTC-Varian Consortium (grant id 2019022) and partly financed by the Surcharge for Top Consortia for Knowledge and Innovation (TKIs) from the Ministry of Economic Affairs and Climate, The Netherlands.

## CRediT authorship contribution statement

**Prerak Mody:** Conceptualization, Methodology, Software, Validation, Formal analysis, Investigation, Data curation, Visualization. **Merle Huiskes:** Methodology, Writing – review & editing. **Nicolas F. Chaves-de-Plaza:** Writing – review & editing. **Alice Onderwater:** Methodology. **Rense Lamsma:** Methodology, Writing – review & editing. **Klaus Hildebrandt:** Writing – review & editing. **Nienke Hoekstra:** Conceptualization, Methodology, Writing – review & editing. **Eleftheria Astreinidou:** Conceptualization, Methodology, Writing – review & editing. **Marius Staring:** Conceptualization, Methodology, Writing – review & editing, Supervision, Funding acquisition. **Frank Dankers:** Conceptualization, Methodology, Software, Data curation, Writing – review & editing, Supervision.

## Declaration of competing interest

The authors declare that they have no known competing financial interests or personal relationships that could have appeared to influence the work reported in this paper.
