# Supplementary material for: Large-scale dose evaluation of deep learning organ contours in head-and-neck radiotherapy by leveraging existing plans
Source: Phys Imaging Radiat Oncol. 2024 Mar 28;30:100572. doi: 10.1016/j.phro.2024.100572 (PMC11021837; doi:10.1016/j.phro.2024.100572)
Supplement: Supplementary data 1 [file mmc1.pdf]

Supplementary Material A. Data Acquisition

The CT scans of our dataset had a dimension of 512 x 512 pixels in the spatial plane with a pixel spacing in the range of [0.92-1.36, 0.92-1.36]mm. Each CT slice was 2mm thick and each scan had between [128,199] slices. The scans were acquired from a Brilliance Big Bore (Philips Healthcare, Ohio, USA) with 120kV and 250mAs. Post acquisition, 64% of patients had Orthopedic Metal Artifact Reduction (O-MAR) processing done.

Supplementary Material B. Automated Contours

The auto-contouring model of RayStation 10B (results in Table B.1 and Table B.2) first performed registration of the chosen CT scan using an atlas of CTs to narrow down CT size so it fits within the graphical processing unit (GPU) used for deep learning. Once registered, the mid-point of each OAR is detected and a 3D bounding box is cropped around that. This cropped area is then passed to a neural net trained for contouring that specific OAR. Each OAR-specific neural net is based on the UNet segmentation architecture whose output is a 3D probabilistic mask for that OAR. As a post-processing step, smoothing is performed on the surfaces of OARs. The model was trained using Tensorflow, an open-source deep neural net software package. During training, rotations, translations and elastic deformations were used to augment the training data. Details on patient cohort were not made public by the manufacturer.

**RoI**	**DICE**	**SDC @ 3mm**	**HD95 (mm)**	**MSD (mm)**
Spinal Cord ($D_{0.03cc}$)	0.78 [0.61,0.93]	0.92 [0.76,0.97]	10.0 [1.1,69.4]	0.9 [0.2,1.4]
Brainstem ($D_{0.03cc}$)	0.70 [0.07,0.95]	0.72 [0.18,0.95]	13.1 [2.5,49.0]	3.1 [1.1,8.3]
Parotid (L) (D_{mean})	0.85 [0.75,0.94]	0.91 [0.78,0.98]	5.0 [2.3,12.3]	1.5 [0.6,3.2]
Parotid (R) (D_{mean})	0.86 [0.74,0.94]	0.92 [0.75,0.98]	4.6 [2.2,15.7]	1.4 [0.6,4.2]
Submand (L) (D_{mean})	0.84 [0.59,0.93]	0.96 [0.74,1.00]	3.1 [1.7,16.3]	1.0 [0.5,5.3]
Submand (R) (D_{mean})	0.85 [0.68,0.92]	0.96 [0.75,1.00]	3.1 [1.7,16.3]	1.1 [0.6,3.5]
Oral Cavity (D_{mean})	0.84 [0.77,0.92]	0.74 [0.59,0.90]	7.7 [4.3,12.0]	2.6 [1.5,3.3]
Larynx (SG) (D_{mean})	0.54 [0.36,0.65]	0.63 [0.51,0.80]	15.9 [7.8,25.0]	5.7 [2.8,10.2]
Esophagus (D_{mean})	0.66 [0.28,0.90]	0.75 [0.41,0.97]	20.4 [2.5,63.9]	1.4 [0.3,18.8]
Mandible (D_{mean})	0.88 [0.81,0.97]	0.94 [0.87,1.00]	4.5 [1.1,14.0]	1.5 [0.2,3.4]

Table B.1: Summary measures (median [5th percentile, 95th percentile]) for volumetric and surface metrics of auto-contours of RayStation 10B.

**RoI**	**DICE**	**SDC @ 3mm**	**HD95 (mm)**	**MSD (mm)**
Spinal Cord ($D_{0.03cc}$)	0.77 [0.74,0.80]	0.89 [0.87,0.91]	19.2 [13.6,24.7]	0.8 [0.7,0.9]
Brainstem ($D_{0.03cc}$)	0.61 [0.61,0.67]	0.66 [0.60,0.72]	18.0 [14.4,21.5]	3.8 [3.3,4.5]
Parotid (L) (D_{mean})	0.84 [0.84,0.86]	0.89 [0.87,0.91]	5.8 [4.8,6.8]	1.7 [1.5,1.8]
Parotid (R) (D_{mean})	0.85 [0.85,0.86]	0.89 [0.87,0.91]	5.8 [4.9,6.9]	1.7 [1.5,2.0]
Submand (L) (D_{mean})	0.80 [0.80,0.84]	0.90 [0.87,0.94]	6.2 [4.3,8.9]	2.3 [1.1,4.3]
Submand (R) (D_{mean})	0.82 [0.82,0.84]	0.92 [0.89,0.94]	4.8 [3.9,5.7]	1.4 [1.1,1.7]
Oral Cavity (D_{mean})	0.84 [0.82,0.86]	0.74 [0.71,0.76]	7.9 [7.2,8.6]	2.6 [2.4,2.9]
Larynx (SG) (D_{mean})	0.51 [0.47,0.54]	0.63 [0.58,0.67]	15.4 [13.7,17.3]	6.1 [5.3,7.0]
Esophagus (D_{mean})	0.66 [0.61,0.70]	0.75 [0.71,0.80]	23.8 [18.6,29.3]	5.8 [4.0,7.8]
Mandible (D_{mean})	0.88 [0.85,0.90]	0.94 [0.92,0.95]	6.1 [4.7,7.6]	1.6 [1.3,1.9]

Table B.2: Summary measures (sample mean [bootstrapped 95% confidence interval]) for volumetric and surface metrics of auto-contours of RayStation 10B.

Supplementary Material C. Automated Planning

For automated planning, we replicated the beam setup, OAR/target objectives for both photon and proton as per our institutions clinical head-and-neck protocol.

For photon (Table C.3), our VMAT plans are made on an isotropic dose grid of 0.2cm The photon beams were commissioned on an Elekta Synergy system with Agility multi-leaf collimator.

For proton (Table C.4), our IMPT plans are made on an isotropic dose grid of 0.3cm. This dose is delivered using pencil beam scanning (PBS) on a Varian ProBeam machine.

**Step**	**RoI**	**Function**	**Description**	**Weight**
1	PTV (DL1)	MinDose	100% of DL1 prescription	80.0 $\rightarrow$ $\{VDT\}$
1	PTV (DL1)	MaxDose	102% of DL1 prescription	50.0 $\rightarrow$ $\{VDT\}$
1	ring $\leq$ PTV (DL1)	MaxDose	96% of DL1 prescription	0.0 $\rightarrow$ $\{VDT\}$
1	PTV (DL2)	MinDose	100% of DL2 prescription	80.0 $\rightarrow$ $\{VDT\}$
1	PTV (DL2)	MaxDose	102% of DL2 prescription	50.0 $\rightarrow$ $\{VDT\}$
1	PTV (DL2)	UniformDose	100% of DL2 prescription	10.0
1	Body	DoseFallOff	From 100% to 0% of DL1 prescription over 5.0 cm	1.0
1	Body	DoseFallOff	From 100% to 26% of DL1 prescription over 2.0 cm	2.0
1	Body	DoseFallOff	From 100% to 64% of DL1 prescription over 0.5 cm	10.0
1	$\text{Ghost}_{\text{Cranial}}$	DoseFallOff	From 100% to 0% of DL1 prescription over 1.0 cm	0.5
1	$\text{Ghost}_{\text{Ear(L)}}$	DoseFallOff	From 100% to 46% of DL1 prescription over 2.0 cm	1.0
1	$\text{Ghost}_{\text{Ear(R)}}$	DoseFallOff	From 100% to 46% of DL1 prescription over 2.0 cm	1.0
1	Brainstem	MaxEUD	eudParameterA=50 (maxEUD=4000 cGy)	3.0
1	Brainstem (+3 cm)	MaxEUD	eudParameterA=50 (maxEUD=4400 cGy)	3.0
1	Spinal Cord	MaxEUD	eudParameterA=50 (maxEUD=4000 cGy)	3.0
1	Spinal Cord (+3 cm)	MaxEUD	eudParameterA=50 (maxEUD=4400 cGy)	3.0
2.1	Other Organs	DoseFallOff	From 100% to 20% of DL1 prescription over 2.0 cm	1.0
2.2	Other Organs	DoseFallOff	From 100% to 0% of DL1 prescription over 2.0 cm (*as determined by treatment planner*)	1.0
3	Other Organs	MaxEUD	eudParameterA=50, maxEUD=$\{VDT\}$	1.0
4	Control Structures	{MinDose, MaxDose}	Dose=$\{VDT\}$	$\{VDT\}$

Table C.3: Our 4-step emulation of the manual photon optimization process of our clinic. In each step, we also optimize for the objectives of the previous steps. We use VDT as an abbreviation for the phrase "value determined by treatment planner". The $\rightarrow$ indicates that the weight is modified at the end of Step 4.. Here DL1/DL2 stands for electives/boost regions of the tumor and prescription refers to a value of cGy that was assigned to a region-of-interest (RoI). Here "Other Organs" refers to Cochlea (L/R), Parotid (L/R). Submandibular (L/R), Muscle Constrictor (S/M/I), Cricopharyngeus, Larynx (SG), Glottic Area, Trachea, Esophagus and Oral Cavity. The rows shown here are created as objectives in our clinic's treatment planning solution.

Step	RoI	Function	Description	Weight	Robust
1	CTV (DL1)	MinDose	100% of DL1 prescription	800.0 $\rightarrow$ {VDT}	$*$
1	CTV (DL1) - (CTV(DL2) + 3 mm)	MaxDose	102% of DL1 prescription	20.0 $\rightarrow$ {VDT}	$*$
1	CTV (DL1) - (CTV(DL2) + 2 cm)	MaxDose	102% of DL1 prescription	80.0 $\rightarrow$ {VDT}	$*$
1	CTV (DL2)	MinDose	100% of DL2 prescription	800.0 $\rightarrow$ {VDT}	$*$
1	CTV (DL2)	MaxDose	100% of DL2 prescription	50.0 $\rightarrow$ {VDT}	$*$
1	CTV (L)	MinDose	0 cGy and Beam={1,2,3}	0.0	
1	CTV (R)	MinDose	0 cGy and Beam={4,5,6}	0.0	
1	Body	DoseFallOff	From 101% to 0% of DL2 prescription over 2.0 cm	1.0	
1	Body	MaxDose	67% of DL2 prescription for each beam	10000.0	
1	Body	MaxDose	107% of DL2 prescription	100.0	$*$
2	Mandible	MaxDose	107% of DL2 prescription	500.0 $\rightarrow$ {VDT}	$*$
2	Organ Set 1	DoseFallOff	From 101% to 0% of DL2 prescription over 2.0 cm	1.0	
2	Organ Set 2	DoseFallOff	From 101% to 0% of DL2 prescription over 2.0 cm	1.0	
3.1	Organ Set 2	MaxEUD	eudParameterA=1, maxEUD={VDT}	1.0	
3.2	Organ Set 2 - (CTV (DL1) + 3 mm)	MaxEUD	eudParameterA=1, maxEUD={VDT}	1.0	
4	Control Structure	{MinDose, MaxDose}	Dose={VDT}	{VDT}	{$*$}

Table C.4: Our 4-step emulation of the manual proton optimization process of our clinic. In each step, we also optimize for the objectives of the previous steps. We use VDT as an abbreviation for the phrase "value determined by treatment planner". The $\rightarrow$ indicates that the weight is modified at the end of Step 4.. Here DL1/DL2 stands for elective/boost regions of the CTV and prescription refers to a value in cGy that was assigned to a region-of-interest (RoI). "Organ Set 1" refers to Mandible, Brainstem, Spinal Cord, Esophagus, Trachea, Larynx (SG), Trachea and Glottic Area, while "Organ Set 2" refers to Parotid (L/R), Submandibular (L/R), Muscle Constrictor (S/M/I), and Oral Cavity. The $*$ mark is used to indicate those objectives which are robustly optimized. The rows shown here are created as objectives in our clinic's treatment planning solution.

Supplementary Material D. Organ Dose Metrics

We show dose metrics for organs available in the RayStation 10B auto-contouring module for photon (Table D.5 and D.6) and proton (Table D.7 and D.8). For the purpose of our study, we only included organs with available auto-contours, although additional organs-at-risk are evaluated clinically.

**RoI**	$\lvert P_{OG} - P_{MC} \rvert$	$\lvert P_{MC} - P_{AC} \rvert$
Spinal Cord ($D_{0.03cc}$)	1.45 [0.06,5.51]	1.13 [0.18,5.16]
Brainstem ($D_{0.03cc}$)	1.88 [0.05,6.77]	2.17 [0.21,6.37]
Parotid (L) (D_{mean})	0.12 [0.02,0.72]	0.32 [0.02,2.10]
Parotid (R) (D_{mean})	0.13 [0.01,0.68]	0.42 [0.03,1.66]
Submand (L) (D_{mean})	0.27 [0.02,1.20]	0.45 [0.05,2.37]
Submand (R) (D_{mean})	0.21 [0.01,1.28]	0.35 [0.04,1.80]
Oral Cavity (D_{mean})	3.24 [0.01,0.86]	0.35 [0.05,1.32]
Larynx (SG) (D_{mean})	0.39 [0.03,1.47]	0.39 [0.21,4.24]
Esophagus (D_{mean})	0.24 [0.01,1.64]	0.65 [0.04,3.43]
Mandible ($D_{2\%}$)	0.37 [0.03,3.43]	0.43 [0.06,2.12]

Table D.5: Median [5th percentile, 95^{th}percentile] of the absolute dose metric values (in Gy) for $P_{OG} - P_{MC}$ and $P_{MC} - P_{AC}$ in photon radiotherapy.

**RoI**	$\lvert P_{OG} - P_{MC} \rvert$	$\lvert P_{MC} - P_{AC} \rvert$
Spinal Cord ($D_{0.03cc}$)	2.01 [1.51,2.56]	1.90 [1.49,2.32]
Brainstem ($D_{0.03cc}$)	2.43 [1.90,3.01]	2.82 [2.36,3.34]
Parotid (L) (D_{mean})	0.21 [0.15,0.28]	0.66 [0.49,0.85]
Parotid (R) (D_{mean})	0.21 [0.15,0.27]	0.62 [0.48,0.80]
Submand (L) (D_{mean})	0.39 [0.30,0.49]	0.80 [0.52,1.22]
Submand (R) (D_{mean})	0.33 [0.23,0.45]	0.59 [0.42,0.80]
Oral Cavity (D_{mean})	0.32 [0.24,0.42]	0.49 [0.40,0.58]
Larynx (SG) (D_{mean})	0.55 [0.39,0.74]	1.65 [1.25,2.07]
Esophagus (D_{mean})	0.41 [0.29,0.54]	1.05 [0.80,1.38]
Mandible ($D_{2\%}$)	0.81 [0.48,1.22]	0.97 [0.54,1.60]

Table D.6: Sample mean [bootstrapped 95% confidence interval] of the absolute dose metric values (in Gy) for $P_{OG} - P_{MC}$ and $P_{MC} - P_{AC}$ in photon radiotherapy.

RoI	$\lvert P_{OG} - P_{MC} \rvert$	$\lvert P_{MC} - P_{AC} \rvert$
Spinal Cord ($D_{0.03cc}$)	2.08 [0.03,8.82]	0.70 [0.12,2.40]
Spinal Cord ($D_{0.03cc}$) (vw-max)	1.90 [0.05,8.07]	0.72 [0.15,2.57]
Brainstem ($D_{0.03cc}$)	0.72 [0.05,3.79]	0.59 [0.03,2.77]
Brainstem ($D_{0.03cc}$) (vw-max)	0.98 [0.13,4.30]	1.00 [0.19,2.81]
Parotid (L) (D_{mean})	0.10 [0.02,0.39]	0.48 [0.07,1.99]
Parotid (R) (D_{mean})	0.14 [0.01,0.43]	0.40 [0.03,1.80]
Submand (L) (D_{mean})	0.21 [0.06,0.79]	0.28 [0.05,1.85]
Submand (R) (D_{mean})	0.18 [0.03,0.70]	0.27 [0.01,1.89]
Oral Cavity (D_{mean})	0.08 [0.02,0.39]	0.31 [0.03,0.73]
Larynx (SG) (D_{mean})	0.37 [0.01,1.36]	0.56 [0.19,3.26]
Esophagus (D_{mean})	0.31 [0.01,3.03]	0.23 [0.07,0.77]
Mandible ($D_{2\%}$)	0.44 [0.01,2.19]	0.79 [0.06,2.92]
Mandible ($D_{2\%}$) (vw-max)	0.52 [0.01,2.98]	0.46 [0.08,2.13]

Table D.7: Median [5th percentile, 95^{th}percentile] of the absolute dose metric values (in Gy) for $P_{OG} - P_{MC}$ and $P_{MC} - P_{AC}$ in proton radiotherapy.

RoI	$\lvert P_{OG} - P_{MC} \rvert$	$\lvert P_{MC} - P_{AC} \rvert$
Spinal Cord ($D_{0.03cc}$)	2.92 [1.93,4.00]	0.92 [0.65,1.20]
Spinal Cord ($D_{0.03cc}$) (vw-max)	2.93 [1.92,4.06]	1.08 [0.79,1.40]
Brainstem ($D_{0.03cc}$)	1.07 [0.67,1.54]	0.89 [0.60,1.20]
Brainstem ($D_{0.03cc}$) (vw-max)	1.35 [0.90,1.84]	1.27 [0.92,1.70]
Parotid (L) (D_{mean})	0.16 [0.11,0.21]	0.63 [0.43,0.87]
Parotid (R) (D_{mean})	0.15 [0.11,0.20]	0.62 [0.41,0.86]
Submand (L) (D_{mean})	0.32 [0.20,0.47]	0.51 [0.32,0.73]
Submand (R) (D_{mean})	0.27 [0.18,0.37]	0.71 [0.29,1.41]
Oral Cavity (D_{mean})	0.15 [0.10,0.21]	0.34 [0.26,0.42]
Larynx (SG) (D_{mean})	0.59 [0.39,0.83]	0.88 [0.54,1.30]
Esophagus (D_{mean})	0.75 [0.42,1.19]	0.34 [0.25,0.45]
Mandible ($D_{2\%}$)	0.88 [0.49,1.40]	1.00 [0.69,1.34]
Mandible ($D_{2\%}$) (vw-max)	0.95 [0.58,1.36]	0.79 [0.54,1.08]

Table D.8: Sample mean [bootstrapped 95% confidence interval] of the absolute dose metric values (in Gy) for $P_{OG} - P_{MC}$ and $P_{MC} - P_{AC}$ in proton radiotherapy.

Supplementary Material E. NTCP

For NTCP scores (Table E.9 and E.10), we used the formulae and parameters from the National Indication Protocol for Proton therapy (*Landelijk Indicatie Protocol Protonentherapie*) [21]. From this document, we referred to Section 3.3.3 and 3.3.4 for xerostomia and Section 3.4.3 and 3.4.4 for dysphagia. For all four toxicities, we used a baseline score of 0.

	Photon		Proton	
	$\|P_{OG} - P_{MC}\|$	$\|P_{MC} - P_{AC}\|$	$\|P_{OG} - P_{MC}\|$	$\|P_{MC} - P_{AC}\|$
Xerostomia Grade ≥ 2	0.1 [0.0,0.5]	0.3 [0.0,0.9]	0.1 [0.0,0.3]	0.2 [0.0,1.0]
Xerostomia Grade ≥ 3	0.0 [0.0,0.2]	0.1 [0.0,0.3]	0.0 [0.0,0.1]	0.1 [0.0,0.3]
Dysphagia Grade ≥ 2	0.2 [0.0,0.9]	0.2 [0.0,0.6]	0.0 [0.0,0.3]	0.1 [0.0,0.3]
Dysphagia Grade ≥ 3	0.1 [0.0,0.7]	0.1 [0.0,0.5]	0.0 [0.0,0.1]	0.0 [0.0,0.1]

Table E.9: Summary measures (median [5th percentile, 95th percentile]) for ΔNTCP (%) values in photon and proton radiotherapy for $|P_{OG} - P_{MC}|$ and $|P_{MC} - P_{AC}|$.

	Photon		Proton	
	$\|P_{OG} - P_{MC}\|$	$\|P_{MC} - P_{AC}\|$	$\|P_{OG} - P_{MC}\|$	$\|P_{MC} - P_{AC}\|$
Xerostomia Grade ≥ 2	0.2 [0.1,0.2]	0.4 [0.3,0.4]	0.1 [0.1,0.2]	0.3 [0.2,0.5]
Xerostomia Grade ≥ 3	0.1 [0.0,0.1]	0.1 [0.1,0.2]	0.0 [0.0,0.1]	0.1 [0.1,0.2]
Dysphagia Grade ≥ 2	0.3 [0.2,0.4]	0.2 [0.2,0.3]	0.1 [0.1,0.1]	0.1 [0.1,0.1]
Dysphagia Grade ≥ 3	0.2 [0.1,0.3]	0.2 [0.1,0.2]	0.0 [0.0,0.0]	0.0 [0.0,0.0]

Table E.10: Sample mean [bootstrapped 95% confidence interval]) for ΔNTCP (%) values in photon and proton radiotherapy for $|P_{OG} - P_{MC}|$ and $|P_{MC} - P_{AC}|$.

Supplementary Material F. Visual Results

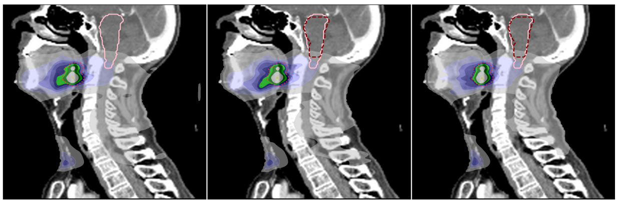

(a) Brainstem (DICE=0.83, $|\Delta D_{0.03cc}| = 5.3\%$)

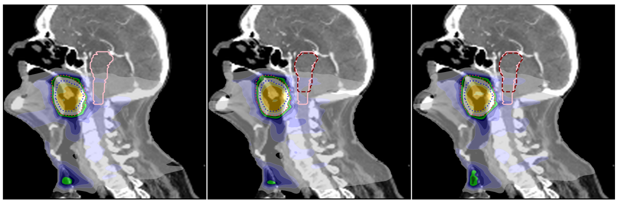

(b) Brainstem (DICE=0.81, $|\Delta D_{0.03cc}| = 28.1\%$)

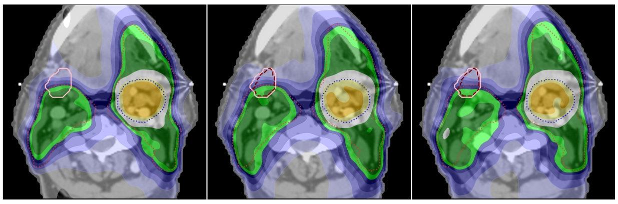

(c) Submand (R) (DICE=0.90, $|\Delta D_{mean}| = 1.4\%$)

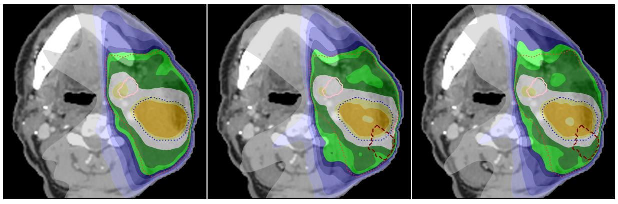

(d) Submand (L) (DICE=0.00, $|\Delta D_{mean}| = 0.5\%$)

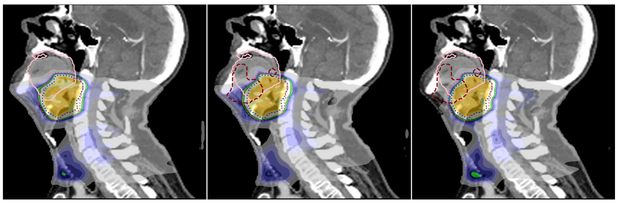

(e) Oral Cavity (DICE=0.42, $|\Delta D_{mean}| = 4.1\%$)

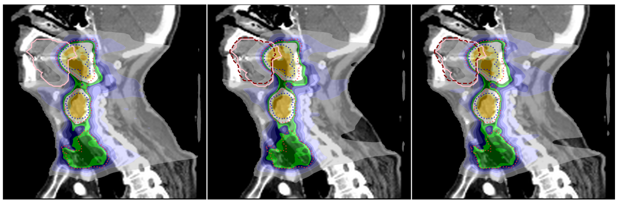

(f) Oral Cavity (DICE=0.87, $|\Delta D_{mean}| = 2.4\%$)

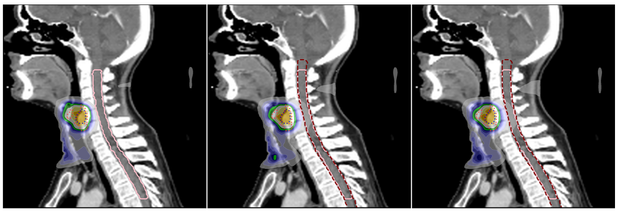

(g) Spinal Cord (DICE=0.80, $|\Delta D_{0.03cc}| = 11.2\%$)

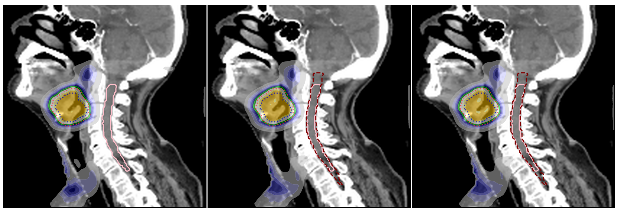

(h) Spinal Cord (DICE=0.57, $|\Delta D_{0.03cc}| = 21.8\%$)

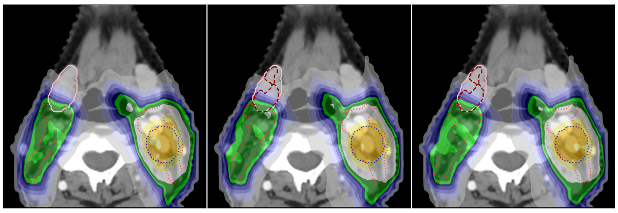

(i) Submand (R) (DICE=0.82, $|\Delta D_{mean}| = 1.3\%$)

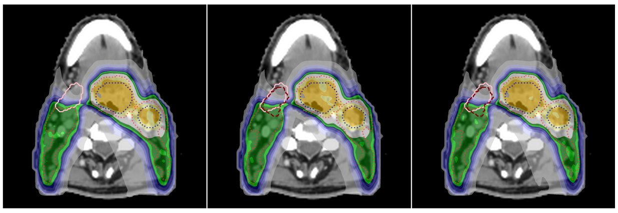

(j) Submand (R) (DICE=0.80, $|\Delta D_{mean}| = 2.6\%$)

Figure F.7: This figure shows CT scans of photon (a-f) and proton (g-j) patients overlayed with a dose distribution as well as PTV (DL1) (orange), PTV (DL2) (blue), manual (pink) and automated (maroon) contours. Each example shows the P_{OG}, P_{MC} and P_{AC} plans from left to right. The dose metric in the sub-captions compares the absolute percentage difference of $P_{MC} - P_{AC}$.
